# Book Reviews

**Published:** 1917-01

**Authors:** 


### BOOK REVIEWS

Books mentioned may be inspected at and ordered through this office. So far as possible, books received in any month will be reviewed in the issue of the second month following.

**Die Schweren Entzuendlichen Erkrankungen des Dickdarmes.** Ad. Schmidt, Halle, Reprint from Archiv fur Verdauungs-Krankheiten, Vol. 22.

**Erkrankungen des Pancreas. Die Funtion Spruefung des Darmes und die Darmdyspepsien. Enteritis und Colitis Acuta und Chronica.** Dr. Ad. Schmidt, Halle, Reprint from Spezielle Pathologie und Therapie innerer Krankheiten.

Issued by the author, not on the market. This is a paper-bound book of 138 large pages, illustrated with 13 colored full-page plates and a number of cuts in the text. Bibliographic references are included.

**Dangers of Induction of Lungcommotion as a Means of Resuscitation.** Dr. Wm. Harrison, Detroit. Pneumatry Papers No. 1. 25 cents.

**Transactions of the American Otological Society.** 49th Annual Meeting. Washington, May 9 and 10, 1916. 201 pages. Published by the Society. Dr. John B. Rae, Sec., 247 W. 70, N. Y.

**40th Annual Report of the Buffalo Eye and Ear Infirmary.** For the period Oct. 1, 1915-June 30, 1916.

This institution has been ranked in Class I by the State Board of Charities for 8 years. For the 9 months, 2230 new cases were admitted: 1383 eye, 346 ear, 445 nose and throat, 56 skin. 10,542 separate consultations were made.

**Medical Clinics of Chicago,** Nov. 1916, Vol. 2, No. 3. Published bi-monthly by W. B. Saunders Co., Philadelphia. About 1200 pages per year, $8.

**Physicians' Visiting List,** P. Blakiston's Son & Co., Philadelphia.

This is the familiar, black-pocket list, dated or undated, ranging from $1.25 for 25 patients per day or week up to $2.50 for 100 patients (2 volumes a year). Monthly editions can also be secured, that is with half cut leaves so that the name is entered every month instead of every week. The special feature announced is the new dose list prepared to correspond to the new U. S. P.

---

**The Operating Room** by Amy Armour Smith, R. N. Formerly Superintendent of New Rochelle Hospital, New York; Superintendent of Nurses at the S. R. Smith Infirmary, Staten Island, and at the Woman's Hospital of the State of New York. 12mo of 295 pages with 57 illustrations. Philadelphia and London: W. B. Saunders Company. 1916. Cloth, $1.50 net.

This seems to be a very thorough review of the problem from the nurse's standpoint, even including a considerable glossary of more or less technical words and list of instruments. Questions of ethics and hospital politics are touched briefly but firmly. The very fact that they are mentioned at all serves to call attention to measures for the eradication of evils. It is unnecessary to advise nurses to use this text book—they will or wont according to their conception of the amount of post-graduate interest which their profession warrants. But many physicians might be surprised at the complexity of the problem from the nurse's standpoint and might derive very valuable hints for their own use.

---

**Personal Health** by William Brady, M. D., Elmira, N. Y. 12mo of 407 pages. Philadelphia and London: W. B. Saunders Company, 1916. Cloth, $1.50 net.

Dr. Brady has not only covered the subject of hygiene but he has illuminated the subject with flashes of wit and he has introduced a good many points of ethics of practical interest to the laity, as the consideration of the quack, the courtesy due the physician, the sad results of teaching a little child to regard the doctor as a bogey. We are not quite prepared to eliminate cold and damp from the etiology of colds though we go most of the way with the author in regarding catarrhal conditions as infections.

---

**Blood-Pressure,** From The Clinical Standpoint by Francis Ashley Faught, M. D. Formerly Director of the Laboratory of Clinical Medicine at the Medico-Chirurgical College, Philadelphia. Second edition, thoroughly revised. Octavo of 478 pages, illustrated. Philadelphia and London: W. B. Saunders Company, 1916. Price, $3.25 net.

We have previously reviewed the first edition and are not surprised that the work has been received so cordially as to require a further edition—and doubtless future ones. The subject is now quite well standardized and no longer in dispute except as to very minor details. Thus, all that we need say is that the author has taken advantage of accumulated experience without having to make material changes in his earlier presentation and that the book is what every practitioner needs as a guide.

---

**Constipation, Obstipation and Intestinal Stasis** by Samuel Goodwin Gant, M. D., LL.D. Professor of Diseases of the Colon, Sigmoid Flexure, Rectum and Anus in the New York Post-Graduate Medical School and Hospital. Second edition enlarged. Octavo of 584 pages, with 258 illustrations. Philadelphia and London: W. B. Saunders Company, 1916. Cloth $6.00 net; Half Morocco $7.50 net.

While the author attacks the problem from the standpoint of the operator, he enters broadly into diagnostic methods and into various non-surgical methods of treatment. Massage and vibratory methods, internal and external hydrotherapy are especially well presented and illustrated. The careful tracing of causes and effects leads to the consideration of most of the intestinal lesions and many others. The preliminary discussion of anatomy, physiology, etc., is excellent, especially the description of the rectal valves with which every one who has had any experience with endoscopy will agree, largely because the author recognizes the considerable variability of these structures. The operations involve many original features and are adequately illustrated.

---

**The Practitioner's Visiting List for 1917.** Four styles: weekly, monthly, perpetual, sixty-patient. Pocket size, substantially bound in leather with flap, pocket, etc.; $1.25. net. Lea & Febiger, Publishers, Philadelphia and New York.

The text portion of The Practitioner's Visiting List of 1917 contains, among other valuable information, a scheme of denition; tables of weights and measures and comparative scales; instructions for examining the urine; diagnostic table of eruptive fevers; incompatibles, poisons and antidotes; directions for effecting artificial respiration; extensive table of doses; an alphabetical table of diseases and their remedies, and directions for ligation of arteries. The record portion contains ruled blanks of various kinds, adapted for noting all details of practice and professional business.

**Medical Directory of N. Y., N. J., and Conn.** Issued by the Medical Society of the State of N. Y., revised to Oct. 10, 1916.

---

As there has been considerable discussion as to the value of this activity of the State Society, proportionable to the very considerable expense, we will state that in the reviewer's opinion, it is one of the most valuable functions which the Society has assumed. There are listed 14,224 physicians for N. Y.; 2,753 for N. J., and 1,502 for Conn.—respective increases over last year of 68, 179, and 41. Greater N. Y. City has 7,781 physicians, a gain of 96 and the rest of N. Y. State 6,443, a loss of 28. It will be seen that the totals represent changes from 1/2 to 7%. The actual changes are considerably greater: 1.5%-2% of the list is lost by death in any average year. As many more names are gained by any average community and local changes of address amount to as much more. At a conservative estimate, the address list of one year is 5% in error by the end of the same year. We feel strongly that it is worth the cost, for the profession to keep track of itself. If the average physician is not interested to this proportionate amount, in knowing who is dead, where his friends and classmates are, what town and college are associated with a familiar name, whether a man who comes to his attention as a stranger, favorably or unfavorably, has a definite status even in the statistic sense, it is not merely a lack of sentiment but a lack of professional solidarity. We urge every physician to use the directory, to keep alive his various professional affiliations with its aid, and to give prompt notice of any changes and errors that he may observe.

---

**A Practical Medical Dictionary.** Thomas Lathrop Stedman, A. M., M. D., N. Y. 4th revised edition, Wm. Wood & Co., limp leather, 1102 pages, $5.

We have previously commented favorably on this dictionary. The present edition follows the third by only two years. More than a hundred new eponyms have been included, the Basle Anatomic Nomenclature has been indicated throughout, even when not differing from the ordinary terms, and a number of new words have been added and various revisions have been made to bring the work thoroughly up to date. It is practical in no apologetic sense, but is a scholarly work, and even the admission of etymologically objectionable terms is usually indicated.

---

**The Healthy Marriage.** G. T. Wrench, M. D., B. S., Dublin.

2d edition, 300 pages, $1.50. Paul B. Hoeber, 69 E. 59, N. Y.

This is a very practical treatise along the lines indicated. It is not so popular as to render it valueless to the physician and not so technical as to prevent its being placed in the hands of the laity.

---

**The Medical Clinics of Chicago.** Volume II, Number III (November 1916). Octavo of 211 pages, 44 illustrations. Philadelphia and London: W. B. Saunders Company. 1916. Published bi-monthly. Price per year: paper $8.00, cloth $12.00.

---

**Physician's Daily Memorandum Book.** Issued free by the M. J. Breitenbach Co., N. Y.

This is the familiar red-covered memory. We have used it for years for business and social engagements, medical meetings, etc., purely professional calls being marked in the regular visiting lists by a dot for advance dates. The loss of money, time, pleasure and experience which would be inevitable without some such reminder, would in the aggregate, be enormous.

---

**N. Y. State Museum Bulletin, No. 187,** published monthly by the University of the State of N. Y. This number consists of about 200 pages, illustrated, including the 69th report of the museum, the 35th of the state geologist, etc. One of the most interesting papers is on recent Landslides in Unconsolidated Sediments.

---

**Annual Report of the Secretary of the Navy, 1916.**

The death rate of the Navy was 4.48:1000, as compared with an average of 8:1000 for men of the same age in civil life. Drowning, tuberculosis and pneumonia are the chief causes of death, the first causing almost exactly 1/6 of the total for the fiscal year—51. Of these, 21 were due to the sinking of the F-4. Of 106,392 applications for enlistment, 30.18% were accepted, as compared with a previous average of 52%, the difference being due to relative excess of applicants. The medical corps now includes 362 officers, the increase to maximum strength authorized by the act of Aug. 29, 1916, requiring 683, as well as 84 dental surgeons.

